# Isolation and characterization of novel reassortant mammalian orthoreovirus from pigs in the United States

**DOI:** 10.1080/22221751.2021.1933608

**Published:** 2021-06-12

**Authors:** Liping Wang, Yan Li, Timothy Walsh, Zhenyu Shen, Yonghai Li, Nirmalendu Deb Nath, Jinhwa Lee, Baoliang Zheng, Ying Tao, Clinton R. Paden, Krista Queen, Shuping Zhang, Suxiang Tong, Wenjun Ma

**Affiliations:** a Department of Diagnostic Medicine/Pathobiology, Kansas State University, Manhattan, KS, USA; b Division of Viral Diseases, Centers for Disease Control and Prevention, Atlanta, GA, USA; c Department of Veterinary Pathobiology, College of Veterinary Medicine, University of Missouri, Columbia, MO, USA; d Department of Molecular Microbiology and Immunology, School of Medicine, University of Missouri, Columbia, MO, USA; e Veterinary Medical Diagnostic Laboratory, College of Veterinary Medicine, University of Missouri, Columbia, MO, USA

**Keywords:** Mammalian orthoreovirus (MRV), swine, reassortant, virus replication and transmission, prevalence

## Abstract

Mammalian orthoreovirus (MRV) infects multiple mammalian species including humans. A United States Midwest swine farm with approximately one thousand 3-month-old pigs experienced an event, in which more than 300 pigs showed neurological signs, like “down and peddling”, with approximately 40% mortality. A novel MRV was isolated from the diseased pigs. Sequence and phylogenetic analysis revealed that the isolate was a reassortant virus containing viral gene segments from three MRV serotypes that infect human, bovine and swine. The M2 and S1 segment of the isolate showed 94% and 92% nucleotide similarity to the M2 of the MRV2 D5/Jones and the S1 of the MRV1 C/bovine/Indiana/MRV00304/2014, respectively; the remaining eight segments displayed 93%–95% nucleotide similarity to those of the MRV3 FS-03/Porcine/USA/2014. Pig studies showed that both MRV-infected and native contact pigs displayed fever, diarrhoea and nasal discharge. MRV RNA was detected in different intestinal locations of both infected and contact pigs, indicating that the MRV isolate is pathogenic and transmissible in pigs. Seroconversion was also observed in experimentally infected pigs. A prevalence study on more than 180 swine serum samples collected from two states without disease revealed 40%–52% positive to MRV. All results warrant the necessity to monitor MRV epidemiology and reassortment as the MRV could be an important pathogen for the swine industry and a novel MRV might emerge to threaten animal and public health.

## Introduction

Mammalian orthoreovirus (MRV) is a double-stranded RNA virus, belonging to the Reoviridae family [1]. The genome of MRVs is approximately 23.5 kb in length and contains 10 segments: three large (L1, L2 and L3), three medium (M1, M2 and M3) and four small (S1, S2, S3 and S4) segments, which encode eight structural proteins (λ1, λ2, λ3, μ1, μ2, σ1, σ2, σ3) and four nonstructural proteins (μNS, μNSC, σNS, σ1s) [2]. The σ1 protein encoded by the S1 segment is responsible for cell-attachment, type-specific antisera neutralization and haemagglutinin activities. Based on the capacity of the σ1 protein, MRVs have been divided into 4 serotypes: type 1 (MRV1) Lang (T1L), type 2 (MRV2) Jones (T2J), type 3 (MRV3) Dearing (T3D) and a putative 4 (MRV4) Ndelle [3,4].

MRVs are widely distributed and are able to infect many mammalian species including humans, pigs, bats, cattle, minks, dogs, cats and civets [5–13]. They are typically believed to cause mild gastroenteritis and respiratory disease which can be either symptomatic or asymptomatic in the infected species [2]. MRVs have been identified in patients with acute respiratory infections and further shown to have potential human-to-human transmission in Malaysia [14,15]. MRV3 is responsible for diarrhoea, fever and respiratory disease in pigs in Asia, Europe and North America [16–20]. Reoviruses have been considered to induce neurological symptoms even though fewer cases of those were reported in contrast to cases of enteric illness and respiratory signs. For example, necrotizing encephalopathy and meningitis induced by MRV2 or MRV3 in infected humans have been documented in Europe and the United States (US) [21,22]. Additionally, both MRV1 and MRV3 can infect the central nervous system (CNS) in mice by different pathways [23,24]. Above evidence indicates that MRVs are likely responsible for more severe diseases other than mild gastroenteritis and respiratory disease in humans and animals.

When multiple lineages of MRVs infect the same host, the segmented nature of MRVs results in reassortment to promote viral evolution and generate novel strains. Reassortant MRV strains have been identified in different animal species including vole, partridge, bat and calf; further studies showed that gene segments of these reassortant MRVs could be from different hosts based on sequence and phylogenetic analysis [12,25–28]. Additionally, a novel orthoreovirus detected in a hospitalized child with acute gastroenteritis showed high similarity to MRVs found in bats in Europe [10]. All facts indicate possible cross-species transmission of MRVs. In this study, we isolated a novel MRV strain from diseased pigs in a US Midwest swine farm in which more than 300 pigs showed neurological signs with approximately 40% mortality. Sequence and phylogenetic analysis revealed that the isolate was a reassortant virus, having the S1 segment from bovine-derived MRV1, the M2 segment from human-derived MRV2 and the remaining eight segments from pig-derived MRV3. Further animal studies showed that the novel MRV isolate was able to infect and cause disease in pigs and transmitted to contact animals.

## Materials and methods

### Ethics statement

The animal experiment to investigate pathogenicity and transmissibility of the MRV isolate in pigs was reviewed and approved by the Institutional Animal Care and Use Committee at Kansas State University and was performed in Biosafety Level 2+ animal facilities under guidance from the Comparative Medicine Group at Kansas State University.

### Pigs

Twenty-one 5-week-old pigs, which were confirmed to be negative to MRV, swine influenza virus (SIV) by real-time RT–PCR targeting M gene [29] and haemagglutination inhibition (HI) against circulating H1 and H3 swine viruses, porcine respiratory and reproductive syndrome virus (PRRSV), porcine epidemic diarrhoea virus (PEDV) [30], transmissible gastroenteritis virus (TGEV) and porcine group A rotavirus [31] by quantitative PCR assays, were used in this study.

### Clinical case

In March 2018, a US Midwest swine farm with approximately 1000 3-month-old pigs experienced a severe disease event, in which more than 300 pigs showed neurological signs “down and peddling” without diarrhoea and 120 pigs died 3–5 days later. Tissue samples including brain, kidney, spleen, lung, liver, heart, intestine and stomach fundus from two euthanized pigs were collected and submitted to Kansas State Veterinary Diagnostic Laboratory for diagnosis.

### Virus isolation and preparation

A 10% tissue homogenate mixture from two pigs was made for routine diagnostics and virus isolation as described previously [32]. Homogenized tissues were filtered and inoculated onto a monolayer of Madin-Darby Canine Kidney (MDCK) cells, which were grown in Minimal Essential Medium (MEM), supplemented with 3% bovine serum albumin (BSA) (Sigma-Aldrich), 1 µg/ml N-tosyl-L-phenylalanine chloromethyl ketone (TPCK)-treated trypsin (Sigma-Aldrich) and 1% antibiotic-antimycotic (Gibco) at 37°C in the atmosphere with 5% CO_2_. The inoculated cells were monitored for cytopathic effects (CPE) every 12 h and blind-passaged for three freeze–thaw cycle passages. Total nucleic acid (NA) was extracted from the supernatant of inoculated cells showing CPE and used for PCR assays and next generation sequencing (NGS).

### Pan-viral group PCR

Pan-viral family/genus PCRs and sequencing were performed for the following viral families/genera: Coronaviridae, Herpesviridae, Orthomyxoviridae (Influenza viruses A, B and C), and Reoviridae (Aquareovirus and Orthoreovirus) [33–36]. First round reverse transcription PCR for RNA viruses was performed with Superscript III/Platinum Taq One Step kits (Invitrogen) and Titanium Taq (Clontech) kits for the second round PCR. First and second round PCR for DNA viruses was performed with Hot Start Ex Taq kits (Takara). A positive PCR control containing mutation-engineered synthetic RNA transcript or DNA amplicon and a negative control using nuclease-free water were included in each run. PCR products were visualized on 2% agarose gels. Positive bands of the expected size that had a strong signal and without additional bands were purified using Exonuclease I (New England Biolabs) and Shrimp Alkaline Phosphatase (Roche). Samples were incubated at 37°C for 15 min followed by 80°C for 15 min to inactivate the Exonuclease and Shrimp Alkaline Phosphatase. Purified PCR amplicons were sequenced with the PCR primers in both directions as described previously [36] on an ABI Prism 3130 Automated Capillary Sequencer (Applied Biosystems) using Big Dye 3.1 cycle sequencing kits (Life Technologies).

### NGS and analysis

Extracted NA was pre-amplified using a modified random amplification protocol as described previously [35]. Briefly, NA samples are reverse-transcribed using a primer containing both a known sequence and a random nanomer, followed by a second primer extension reaction using the same primer [36]. These extension fragments are then amplified by PCR using the known sequence of the extension primer. PCR amplicons obtained from the pre-amplification were purified, fragmented and used to construct libraries with dual index barcoding for Illumina sequencing on a MiSeq instrument as previously described [37].

Initially, reads were assembled and classified using SURPI [38]. Subsequently, reovirus reads identified by SURPI were extracted and further analyzed by de novo assembly as well reference-based assembly with Geneious v11.1.4. Consensus sequences of all genomic segments were generated by Geneious and used for phylogenetic analysis.

### Sequence analysis and characterization of the novel MRV isolate in cells

Each segment sequence of the isolate was blasted and compared with available sequences in GenBank, and the hit with the best identity for each gene was recorded. Each segment sequence of the closely related and other typical reovirus strains were downloaded for further phylogenetic analysis. Maximum likelihood phylogenetic tree of each segment based on the open reading frame was built with MEGA-X using the Hasegawa-Kishino-YanoJukes model with a bootstrap value of 1000. GenBank accession numbers for each segment of this isolate are MW929746-55.

We further used a specific antibody (anti-reovirus capsid protein μ1C monoclonal antibody) to perform immunofluorescence assay (IFA) to detect viral antigens and compared virus replication in different cells including swine testis (ST), human lung adenocarcinoma (A549), and monkey kidney (Marc145) and canine MDCK cells (Supplement materials).

### Pig study design

Twenty-one 5-week-old pigs were used in this study. Nine pigs were inoculated with the MRV isolated in this study (1.0 × 10^7^ TCID_50_/pig in 3 ml) intranasally (1.5 ml through the intranasal route) and orally (1.5 ml through the oral route), and three naïve pigs were commingled with infected pigs at 2 days post infection (dpi) to investigate virus transmission. Another nine pigs were mock-inoculated as controls and housed separately. Clinical signs and body temperature were monitored daily. Rectal swab sample was collected from each pig daily and nasal swabs were collected every two days. Blood samples were collected from infected and mock-infected pigs at 0, 3, 5, 7, 9 and 14 dpi and from contact pigs at 0, 3, 5 and 7 days post contact (dpc). Three infected and control pigs were necropsied at 4 and 7 dpi, three contact pigs were necropsied at 7 dpc. During necropsy, tissue lesions were evaluated by an experienced pathologist; the tissues including duodenum, jejunum, ileum, ileocolic junction, spiral colon, descending colon, brain, lung, kidney, heart, liver and spleen were collected for further virological and histopathological analysis. We developed a RT-qPCR that targeted the conserved region of MRV L1 genes (Supplemental materials, tables and figures) to determine virus loads in collected samples. The remaining three infected and control pigs were kept for 14 days to determine seroconversion.

### Histopathology and immunohistochemistry (IHC) analysis

Tissues collected from each animal in the pig study were fixed in 10% neutral buffered formalin for further histopathological analysis. Based on initial PCR screening and clinical signs, a subset of positive tissues from infected pigs (n = 3) along with matching tissues from the control animal at 4 dpi were routinely processed and stained with haematoxylin and eosin (H&E) and IHC. A board-certified veterinary pathologist evaluated histopathological lesions of each slide of stained tissues in a blinded fashion. IHC was conducted to detect MRV antigens by using the mouse monoclonal antibody against reovirus capsid protein μ1C (10F6; DSHB, USA; 1:80 diluted) and the second antibody Power-Vision Poly-AP anti-mouse IgG.

### HI assay

A total of 228 swine serum samples were collected from three states including Kansas, Texas and Minnesota (Table 2). Serum samples collected from gnotobiotic, caesarian-derived colostrum-deprived piglets without maternal antibodies, which are MRV negative, were used for the negative control. HI assay was performed as described previously with modifications [39]. Briefly, 200 µl receptor destroying enzyme (RDE II) (DENKA SEIKEN, Japan) -treated serum was transferred to 96-well microtiter plates and 10 µl of 25% (v/v) swine RBCs collected from MRV-seronegative pigs was added into each well. After 1-h incubation at 4°C, the 1:10 diluted serum was transferred to a new 96-well plate and added with four haemagglutination units of MRVs. After 1-h incubation at room temperature, 50 ul 1% swine red blood cells from healthy pigs were added into each well and incubated for another 1 h. The HI titer equal to or greater than 20 was judged as positive [39].

## Results

### Initial screening of clinical samples from pigs with neurological signs

After receiving the tissue samples from two diseased pigs with neurological signs, the Kansas State Veterinary Diagnostic Laboratory performed a panel of routine molecular diagnosis, aerobic culture and histopathological analysis. Molecular diagnosis of tissue homogenate mixtures using RT–PCR or RT-qPCR assays showed that both PRRSV and atypical porcine pestivirus were not detected. Results of aerobic culture showed that *Bordetella bronchiseptica* was detected in lungs of one pig and *Staphylococcus* was found in the brain of one pig. Histopathological analysis revealed both pigs had moderate lymphohistiocytic, interstitial pneumonia in the lungs, moderate multifocal atrocytic hypertrophy and swelling with minimal gliosis in the brain and mild, lymphocytic, plasmacytic and eosinophilic enterocolitis in small intestine (Figure S2).

Further analysis showed that tissue homogenates were also negative for influenza A, B, C and D viruses, porcine teschovirus, sapelovirus and encephalomyocarditis virus by RT–PCR or real-time PCR assays.

### Isolation and characterization of a novel reassortant MRV

**Figure 1.**
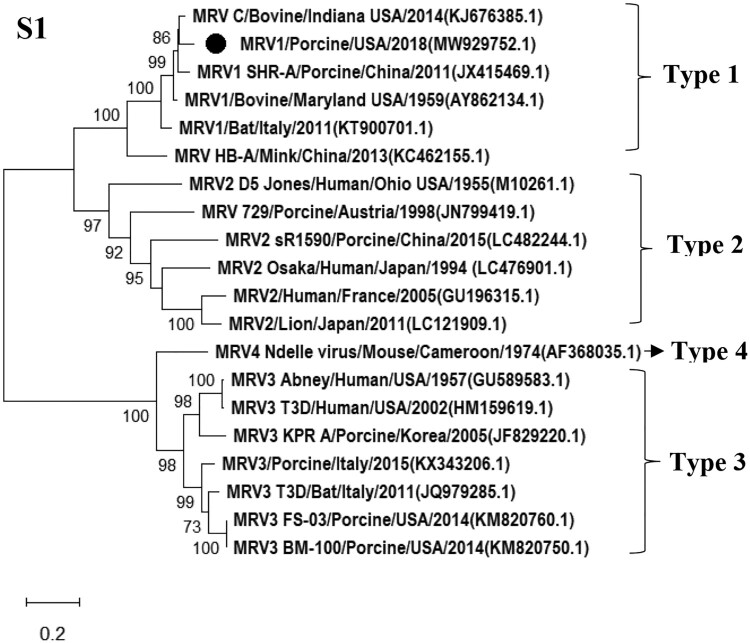
Phylogenetic tree of S1 segment of novel reassortant MRV/USA/Porcine/2018. Related MRV strains were downloaded from GenBank, and open reading fame of each gene segment was used for building the phylogenetic trees. The MRV isolate identified in this study is labelled with a round dot.

CPE in MDCK cells was observed at the third passage of the tissue homogenates from both pigs. Pan-viral group PCR for Reoviridae was positive to MRV and negative to other viral families tested using extracted NAs from the collected supernatant of inoculated cells showing CPE. Results of NGS revealed a novel MRV strain present in the sample, and full genome sequences of all 10 segments were obtained. Sequence analysis showed that the S1 segment displayed 92% sequence identity with a bovine-derived MRV1 (C/bovine/Indiana/MRV00304/2014) detected in bovine calves in the USA in 2014 at the nucleotide level [12], the M2 segment was closely related to the human D5/Jones MRV2 strain (94% homology), while the remaining eight segments were highly homologous to the swine-origin MRV3 (T3/Swine/FS03/USA/2015 and T3/Swine/BM100/USA/2015) detected in pigs in the USA at nucleotide level [19]. The highest nucleotide homology for each segment of the isolate compared to available sequences of MRV strains in GenBank was depicted in Table 1. Phylogenetic tree of S1 segment of the virus was generated and shown in Figure 1, while phylogenetic trees of other segments were shown in Figure S3. Results of sequence and phylogenetic trees indicate that the novel MRV isolate was a novel reassortant among three MRV serotypes (MRV1-3) and was named as the MRV/Porcine/USA/2018 (Table 1, Figures 1 and S3). The S1 segment encodes both σ1 and σ1s, which are responsible for cell-attachment. By comparing with σ1 and σ1s protein sequences of MRV1 available in the GenBank, fourteen substitutions (81A, 118F, 196I, 205D, 249G, 279D, 283N, 285G, 291D, 295 V, 321I, 334 V, 389A, 405I) were identified in the δ1 and one substitution (99H) in the δ1s of this novel MRV/Porcine/USA/2018 isolate. Especially, four substitutions (321I, 334 V, 389A, 405I) were located at the head domain of the σ1 protein that is responsible to bind the MRV serotype-independent receptor – junctional adhesion molecule-A [40,41].

**Table 1.** Highest nucleotide identities of MRV strains with each gene segment of the novel reassortant MRV/Porcine/USA/2018.

Genetic segment	Identity %	MRV strain	Serotype	Host	GenBank No.
L1	94	FS-03/Porcine/USA/2014	3	Pig	KM820754.1
L2	94	T3/Bovine/Maryland/1961	3	Bovine	AF378008.1
L3	95	FS-03/Porcine/USA/2014	3	Pig	KM820756.1
M1	95	FS-03/Porcine/USA/2014	3	Pig	KM820757.1
M2	94	MRV2 D5/Jones	2	Human	M19355.1
M3	94	FS-03/Porcine/USA/2014	3	Pig	KM820759.1
S1	92	C/bovine/Indiana/MRV00304/2014	1	Bovine	KJ676385.1
S2	95	BM-100/Porcine/USA/2014	3	Pig	KM820751.1
S3	94	FS-03/Porcine/USA/2014	3	Pig	KM820762.1
S4	93	FS-03/Porcine/USA/2014	3	Pig	KM820763.1

**Figure 2.**
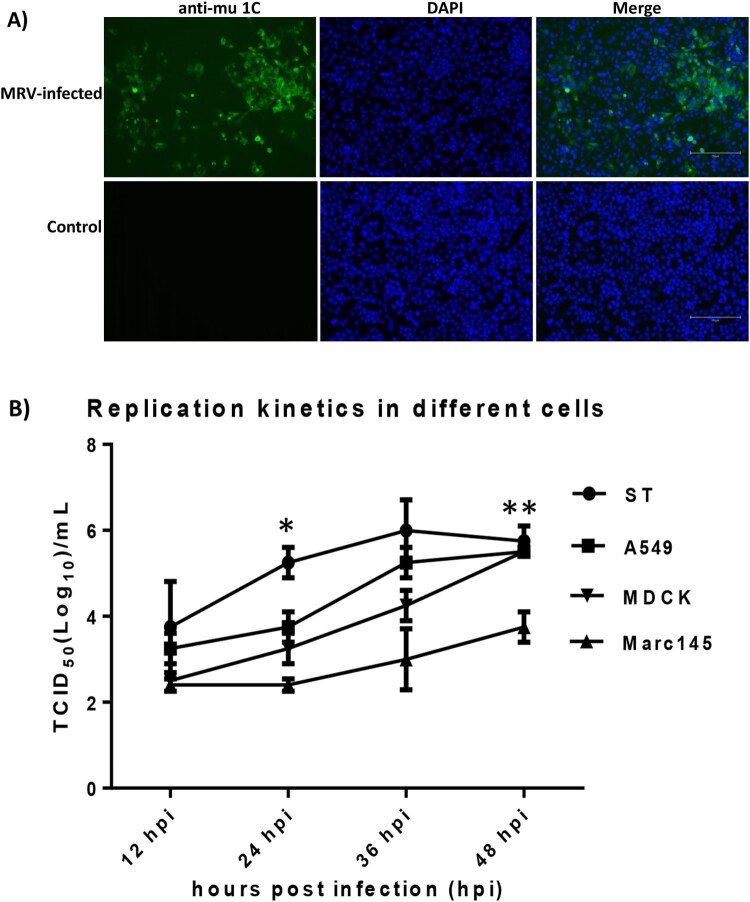
Detection of MRV antigens in infected MDCK cells by IFA and growth dynamics of the MRV isolate in different cells. A) MDCK cells were mock-infected or infected with the novel MRV isolate for 48 h and fixed for IFA assay. The fixed cells were incubated with the anti-mu 1C monoclonal antibody and the FITC-labelled goat anti-mouse IgG second antibody. Nuclei were stained with 4’,6-diamidino-2-phenylindole (DAPI) (Scale bar 150 µm). B): A monolayer of each cell including swine ST, human A549, canine MDCK and monkey Marc145 cells was infected with the MRV isolate at a multiplicity of infection (MOI) of 0.05 TCID_50_. The data points of the curves indicate mean ± SD (*n* = 2). The asterisks (*) represent a statistically significant difference between groups (*p <* *0.05, **p <* *0.001, unpaired *t* test).

The MRV isolate was able to replicate efficiently and grew to titer of 8 log_10_ TCID_50_ per mL in MDCK cells supplemented with TPCK-trypsin (1 µg/mL). The MRV antigen was visualized in the cytoplasm of infected MDCK cells by using an anti-reovirus μ1C protein monoclonal antibody 10F6 (Figure 2A). Additionally, we compared virus replication of the MRV isolate in different cells from different species. Results showed this virus was able to replicate efficiently in human A549, canine MDCK and swine ST cells; however, this virus grew a lower titer in monkey Marc145 cells in contrast to other three cell lines (Figure 2B).

### Pathogenicity and transmissibility of MRV in pigs

Pigs in the mock-infected group did not show clinical signs throughout the length of the study. Clinical signs including fever, diarrhoea and nasal discharge were observed in both infected and contact pigs. Six out of nine infected pigs displayed fever (over 40°C) starting at 2, 3 or 4 dpi, lasting for 2–3 days, and six out of nine infected pigs showed diarrhoea starting at 1 or 4 dpi, lasting for 1–4 days. One out of nine infected pigs showed “walking discordant” (a neurological sign), starting at 2 dpi and lasting for 3 days, and two out of nine infected pigs showed nasal discharge, starting at 6 dpi and lasting for 2 days. Notably, two out of three contact pigs developed fever, starting at 1 or 5 dpc and lasting for 1 day, and two out of three contact pigs had diarrhoea, starting at 3 or 6 dpc and lasting for 2–3 days; one out of three showed nasal discharge, starting at 4 dpc and lasting for 2 days.

**Figure 3.**
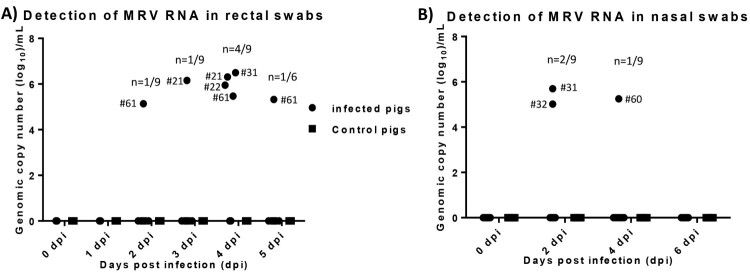
Viral RNA detection in both rectal and nasal swab samples collected from infected and control pigs. A) MRV RNA copy number detected in rectal swab samples collected from infected and control pigs. B) MRV RNA copy number detected in nasal swab samples collected from infected and control pigs.

We employed the developed RT-qPCR_L1 to determine virus loads in collected samples during the pig study. No MRV viral RNA was detected in both nasal and rectal swab samples collected from control or contact pigs. MRV viral RNA was detected in rectal swab samples collected from one infected pig (#61) at 2, 4 and 5 dpi, and from one infected pig (#21) at 3 and 4 dpi and from another two infected pigs (#22 and #31) at 4 dpi (Figure 3A). MRV viral RNA was detected in nasal swabs from two infected pigs at 2 dpi and from another infected pig at 4 dpi (Figure 3B). Rectal and nasal swab samples positive to MRV was only found in one infected pig (#31).

MRV viral RNA was only detected in the homogenate of intestine, not in other tissues collected from both infected and contact pigs. Two infected pigs necropsied at 4 dpi and two contact pigs necropsied at 7 dpc showed MRV viral RNA positive in their intestine tissues. Viral RNA of 10^5.20^ molecules (per gram) was detected in the duodenum sample from one infected pig (#60) whose nasal swabs were positive to MRV at 4 dpi, while the intestine samples including jejunum, colon and ileum collected from another infected pig (#30) were positive to MRV with a titer of 10^7.69^, 10^6.80^ and 10^7.93^ RNA molecules per gram. In contrast, viral RNA of 10^5.32^ molecules per gram was detected in the duodenum sample of one contact pig (#64), while the colon sample from another contact pig (#49) was positive to MRV with a titer of 10^7.82^ RNA molecules per gram. All infected pigs seroconverted at 7 dpi with HI titer ranging from 40 to 320 (the geometric mean was 71), and the HI titer increased at 9 and 14 dpi (the geometric mean was 101 and 202 at 9 and 14 dpi, respectively). However, no HI titer was detected in contact and control pigs.

**Figure 4.**
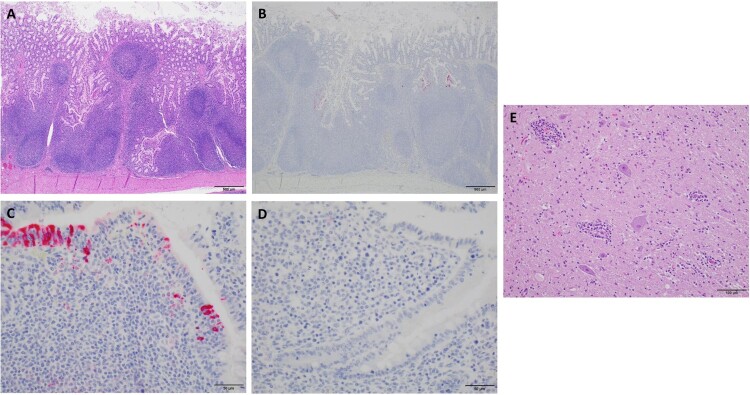
Analysis of sections of intestine and brain of infected and control pigs by H&E and IHC. A) H & E stain with prominent lymphoid follicular development in an infected pig (Scale bar, 500 μm). B) IHC for MRV with strong positive staining of follicular associated epithelium (FAE) and underlying lymphoid tissue in an infected pig. No staining noted in lymphoid follicles proper (Scale bar, 500 μm). C) The image represents segments of FAE overlying lymphoid follicles in the terminal ileum of an infected pig. Staining was consistently present on the lower lateral aspect of the FAE with mild staining in the lamina propria between the epithelium and underlying lymphoid follicle proper (Scale bar, 50 μm). D) The image represents segments of FAE overlying lymphoid follicles in the terminal ileum of in a control pig (Scale bar, 50 μm). E) H & E stain of the section from base of cerebellum inoculated pig #20. Prominent perivascular cuffs of lymphocytes, macrophage-like cells, and rare plasma cells. There is mild diffuse gliosis. In other areas, distinct foci of gliosis are prominent (Scale bar, 100 μm).

Two of three infected pigs which were necropsied at 4 and 7, respectively, had large and swollen mesenteric lymph nodes in contrast to control pigs. Other organs had no obvious changes in infected pigs compared to those of control pigs. Based on the positive results of initial PCR screening and clinical signs, the intestine sections from duodenum, mid jejunum and ileum and ileocecocolic junction, and bilateral sections of rostral cerebrum, thalamus, hippocampus, midbrain (colliculi), brainstem (obex, cerebral peduncles) and cerebellum from three infected pigs and three control animals necropsied at 4 dpi were further examined. All intestine sections of three infected pigs had missing or exfoliating villous tip epithelium with mild to moderate populations of lymphocytes and plasma cells and multifocally neutrophils in lamina propria (Figure 4A). However, no significant difference was observed when compared to intestinal sections from the control pigs, suggesting that pigs used were likely infected during the experiment or already infected by unknown pathogens prior to the experiment. IHC staining showed that one (pig #30) of three infected pigs had strong staining of follicle associated epithelium (FAE) within the ileum accompanied by mild to moderate staining of the underlying lymphocytes in lamina propria, while one infected pig (#60) had weak staining of FAE. No staining was observed in three control and other infected pigs. There was no staining of epithelium away from ileal Peyer’s patch or in adjacent lymph nodes in all infected and control pigs (Figure 4B–D).

In sections of the brain, one inoculated animal (pig #20) with clinical neurological signs described as “walking discordant”, had mild to moderate, multifocal, perivascular cuffs of lymphocytes, rare plasma cells, and macrophages along with scattered minimal to moderate collections of irregular glial cells (gliosis) throughout the thalamus, midbrain, and base of cerebellum (Figure 4E). However, the results of the IHC staining were negative for any sections of brain of this pig and other two infected animals as well as control pigs. The result of the pig study was summarized in Table S2.

### Sero-prevalence of MRV in pigs with different ages

To determine the sero-prevalence of MRV in pigs, a total of 228 serum samples were collected from pigs at Kansas, Texas, and Minnesota and tested by the HI assay using the novel isolate MRV/Porcine/USA/2018. The Kansas swine farm had experienced an outbreak of neurological disease while no obvious clinical signs was observed in pigs from both Texas and Minnesota. Ninety-six percent of serum samples collected from Kansas 3-month-pigs were positive to MRV, while serum samples from post-weaned 3-week-piglets from Texas and Minnesota in 2014 and 2018 were 40% and 52% positive to this virus, respectively (Table 2). Notably, serum samples collected in Minnesotan pigs without disease had the highest of geometric mean HI titer. The results suggest the MRV is likely widespread in swine herds.

**Table 2.** MRV sero-prevalence in pigs at different geographical regions in the US.

	Pig age	No. of samples	Positive Percent (positive number/total number)	HI titer	Geometric Mean	Collection Time
Kansas	3 months	47	96% (45/47)	20–320	52.0	2018
Minnesota	3 weeks	111	52% (58/111)	20–160	66.1	2018
Texas	3 weeks	70	40% (28/70)	20–80	27.6	2014

## Discussion

In this study, we isolated a novel MRV/Porcine/USA/2018 from pigs in US Midwest swine farm in which approximately 300 pigs displayed neurological signs with approximately 40% mortality. Sequence and phylogenetic analysis revealed that this isolate was a reassortant strain among three MRV serotypes (MRV1-3). The segmented nature of MRVs leads to reassortment among different serotypes or strains to produce novel viral strains. One former study showed that a novel reassortant bat MRV virus had the S1 gene similar to those from the bovine-derived MRV1 viruses and other remaining genes from bat MRV viruses [27] and another study revealed that MRV isolates in different bat species in China were closely related to human, swine and mink orthoreoviruses [7]. The novel reassortant MRV isolate reported in this study provides further evidence on reassortment among three MRV serotypes. Although we used the mixed tissues from two pigs to perform virus isolation on MDCK cells, only the single and same MRV sequence was detected in both originally submitted tissues and amplified viruses, indicating that the same virus was circulating in the farm and viral reassortment on MDCK cells was likely not possible. However, how this novel virus was generated and whether an intermediate host was needed remains unknown and needs to be investigated. Importantly, bat-origin orthoreoviruses such as Melaka virus and Kampar virus have been associated with human infections [14,15,42]. The MRV3 has been reported to infect human and multiple animal hosts and was recently identified in alpine chamois [43]. In addition, a novel orthoreovirus that causes acute gastroenteritis in a hospitalized child has been isolated in Europe and revealed that the virus most likely originated from bats [10]. All these results demonstrate interspecies transmission and frequent reassortment events of MRVs.

Our pig studies indicate that the MRV isolate is pathogenic and transmissible in pigs, evidenced by disease, and virus replication and transmission found in both infected and contact pigs. Noticeably, only one of nine infected pigs showed a neurological sign “walking discordant”, while most infected and contact pigs had diarrhoea. Encephalitis was histologically observed in the brain of this pig although MRV antigens were not detected, it could be due to low virus amount and a short period of infection (4 dpi). MRV RNA was detected in both rectal and nasal swabs collected from infected pigs, indicating of fecal-oral transmission routes that is consistent with the previous finding [44]. No MRV RNA was detected in both rectal and nasal swabs from contact pigs while viral RNA was found in intestine tissues from two of three contact animals, suggesting that the MRV transmissibility is low, and it might not maintain in swine herds. All infected pigs seroconverted starting at 7 dpi while none of the contact animals seroconverted, suggesting that most likely it is due to the low virus amount obtained from the infected pigs and a short time incubation. MRV antigens were detected in the FAE in the Peyers patch of the ileum of infected pigs with diarrhoea, which corresponded to the reported M cell distribution in pigs [45] and staining patterns seen in mouse reovirus experiments [46], suggesting the MRV adheres to the same cells to start replication in different species. Results of our pig study are not consistent with the neurological disease with high mortality observed in diseased pigs in the outbreak farm. In addition, viral RNA and antigens were only detected in the intestine rather than brain of infected pigs in our study. This discrepancy suggests that other factors such as unknown pathogens, environmental factors and stress, might be needed to reproduce the disease. To find out whether other pathogen(s) may be responsible for the outbreak in the farm, we performed deep sequencing on the homogenates of the original swine tissues. Viral sequences identified with high significance in the original swine tissue homogenates included porcine parvovirus and porcine adenovirus and 27 different bacterial species (Supplemental results). In addition, only one pair of MRV-specific reads that showed 100% sequence identity to the pig MRV/Porcine/USA/2018 isolate in the RNA library, confirming the presence of the pig MRV with very low virus load in swine tissues, which is consistent with the fact that it took three passages to successfully isolate the MRV in cell cultures. Based on our knowledge, the identified porcine parvovirus and bacterial species are not related to neurological diseases in pigs and they might be as a co-factor to cause the outbreak in the farm. Since porcine adenoviruses have been associated with encephalitis in addition to respiratory and/or enteric disease [47–49], the porcine adenovirus could play a critical role in this outbreak. Further studies are needed to understand whether porcine adenovirus or multiple pathogens such as MRV, porcine adenovirus and/or other co-factors in combination are required to reproduce the neurological disease observed in pigs.

Encephalitis and meningitis caused by MRV infection have been documented in infected humans [21,22]. The pathway of MRV infection of CNS in newborn mice has been systemically investigated. Serotype 1 reovirus spreads to CNS via the haematogenous route, resulting in self-limiting hydrocephalus, while serotype 3 strain enters CNS by neutral routes and causes lethal encephalitis [24,50]. Further studies to investigate dissemination pathways and neural tropism using serotype 1 and 3 reassortant clones suggest that reovirus virulence in CNS may be related to specific interactions between haemagglutinin and neuronal surface receptor [51–53]. Based on our knowledge, the novel MRV strain described in this study is the second isolate associated with neurological disease in pigs. The first MRV related to neurological disease was an MRV2 virus which was isolated from a pig with encephalitis in Austria. In contrast to two former swine MRV3 stains identified in the U.S in 2015 that caused diarrhoea in piglets [19], the novel MRV isolate has a different S1 and M2 gene, suggesting that they might be responsible for inducing different phenotypes of disease in pigs. Further studies are needed to investigate their roles in virus pathogenicity and tissue tropism using reverse genetics.

We have shown a high MRV sero-prevalence in pigs in the US at different ages, suggesting the MRV could be widespread and an important pathogen for swine industry. A previous study showed that there was a very high MRV sero-prevalence in infants in Nashville, Tennessee, likely related to maternal antibody because seropositive rates could be up to 75% in 0–3-month-old infants and 11% in 3–6-month-old babies, while it decreased to 0% in children at 6–12 months of age [54]. A range of 40%–52% sero-positivity in post-weaned (3-week-old) piglets at two different states suggest that the MRV-seropositive could also be associated with maternal antibodies. However, the Minnesota farms had a much higher positive rate than the Texas herds for the same-age pigs tested; moreover, the former one had the highest geometric mean HI titer than the outbreak farm in Kansas, suggesting that real infections occurred in these herds. Noticeably, 3–6-month-old pigs in the disease outbreak farm in this study were 96% MRV-seropositive, suggesting that MRV outbreak in this farm might be responsible for the severe infections in pigs. Additionally, previous studies have revealed that MRV3 σ1-based indirect ELISA assay can also detect MRV serotype 1 strains [55] and the feline MRV cross-reacts with three MRV serotypes based on the neutralization testing [39], suggesting a potential serological cross-reaction among different serotypes of MRVs. Therefore, whether the high sero-prevalence in pigs we found is MRV1-specific or due to cross-reactivity with other serotypes, or due to maternal antibodies needs to be determined in future studies.

In conclusion, we isolated and characterized a novel reassortant MRV virus that was pathogenic and transmissible in pigs although we did not reproduce the neurological disease in experimentally infected pigs. Our results combined with previous studies indicate that MRV is an important pathogen for the swine industry [19]. Therefore, further surveillance and pathogenicity studies on MRVs in pigs should be performed to understand viral pathogenicity and transmissibility as well as reassortment of MRVs as the novel reassortant MRV might emerge to threaten animals and public health.

## Supplementary Material

Supplemental_materials_and_results_Final_05132021.docx
