# Supplementary material for: Isolation and characterization of novel reassortant mammalian orthoreovirus from pigs in the United States
Source: Emerg Microbes Infect. 2021 Jun 12;10(1):1137–47. doi: 10.1080/22221751.2021.1933608 (PMC8205024; doi:10.1080/22221751.2021.1933608)
Supplement: Supplemental_materials_and_results_Final_05132021.docx [file TEMI_A_1933608_SM3337.docx]

Isolation and characterization of novel reassortant mammalian orthoreovirus from pigs in the United States

Liping Wang^1,3,4#^, Yan Li^2#^, Timothy Walsh^1^, Zhenyu Shen^3,5^, Yonghai Li^1^, Nirmalendu Deb Nath^1^, Jinhwa Lee^1^, Baoliang Zheng^1§^, Ying Tao^2^, Clinton R. Paden^2^, Krista Queen^2^, Shuping Zhang^3,5^, Suxiang Tong^2^, Wenjun Ma^1,3,4^*

Supplemental materials and data

**Materials and Methods**

***Immunofluorescence Assay (IFA)***

MDCK cells was infected with the isolated MRV, and then fixed with 10% methanol for 10 min at 48 hours post infection. After one hour incubation with 5% fetal bovine serum (FBS), the cells were incubated with anti-reovirus capsid protein μ1C monoclonal antibody (10F6; DSHB, USA) at a 1:50 dilution at room temperature for 2 hours. The cells were then washed for 3 times with phosphate buffered saline with tween 20 (PBST) and incubated with the second antibody FITC-labeled goat anti-mouse IgG (H+L) (Jackson ImmunoResearch, USA) with 1:200 dilution for 2 hours. After washing the cell monolayer 3 times with PBST, fluorescence was observed under the microscope. Uninfected cells were processed in parallel and used as negative control.

***Growth curve assays***

Swine testis cells (ST), human lung adenocarcinoma cells (A549), monkey kidney cells (Marc145) were grown in Dulbecco's Modified Eagle Medium (DMEM) supplemented with 10% FBS and 1% antibiotics, while MDCK cells were grown in MEM supplemented with 5% FBS and 1% antibiotics at 37℃ in the atmosphere with 5% CO2. A monolayer of each cell was infected with the MRV isolate at a multiplicity of infection (MOI) of 0.05 TCID_50_/cell in the infection medium containing 3% BSA with the presence of 1 ug/ml TPCK-trypsin. Viral titers in each infected cell were determined at 12, 24, 36 and 48 hours post infection (hpi) on MDCK cells by using IFA as described above.

***MRV real-time RT-PCR assay***

To detect all serotypes of MRVs, a quantitative real-time PCR (RT-qPCR_L1) was developed by targeting the conserved region of MRV L1 genes based on sequence information available in GenBank. The probe was labelled with 6-carboxyfluorescein and with 6-carboxytetramethylrhodamine at the 5’ and 3’ ends, respectively; and nucleotide information of primers and probe is summarized in Supplemental Table 1. The RT-qPCR_L1 assay was performed by using qScript XLT 1-Step RT-qPCR ToughMix (Quantabio, Beverly, Massachusetts, USA) according to the manufacturer’s instructions with a total reaction system of 10 µl, which contained 5 µl of ToughMix, 0.48 µl of L1-F (12 µM), 0.48 µl of L1-R (12 µM), 0.16 µl of L1-Probe (4 µM), 1.38 µl of H_2_O, and 2.5 µl of RNA template. The thermos-cycling conditions were set as follows: 50°C for 10 min, 95°C for 1 min, then 45 cycles of at 95°C for 10 seconds and 60°C for 45 seconds. The analytic sensitivity of the RT-qPCR_L1 assay was assessed using 10-fold serially diluted in vitro-transcribed RNA of the MRV isolate L1 in duplicate repeats, ranging from 1x 10^-2^ to 1 x 10^9^ RNA molecules by two independent experiments according to above conditions. In addition, RNA samples extracted from the serially diluted MRV isolate with a known titer were also tested to determine sensitivity of the developed assay. The specificity of this assay was determined by testing swine pathogens including SIV, PRRSV, TGEV and PEDV.

***Next generation sequencing for*** ***swine clinical tissue homogenate***

To maximize the chance to capture different types of sequences, RNA and DNA of the sample were extracted separately and then subjected to three different library protocols to target RNA, ssDNA, and dsDNA respectively. Briefly, swine tissue homogenate was ultracentrifuged, and DNA (DNeasy Blood & Tissue Kit) and RNA (QIAamp Viral RNA Mini Kit) were extracted using commercial kits based on the manufacture instructions. Libraries for Illumina sequencing from RNA and dsDNA were prepared using Zymo-Seq RiboFree Total RNA Library Kit (Zymo) and Illumina DNA Prep Kit (Illumina), respectively, by following manufacturer’s instructions. The library for ssDNA sequencing was prepared by converting ssDNA in the extracted DNA to dsDNA and then following the Illumina DNA Prep Kit protocol. Prior to sequencing the libraries were quantified using Qubit dsDNA HS Assay Kit (ThermoFisher Scientifc) and subjected to fragment analysis in DNA Core of University of Missouri-Columbia. The libraries were sequenced individually with MiSeq V3 (2 x 300 bp) (Illumina).

***Sequencing data processing***

Sequencing data were processed using CLC Genomics Workbench 21.0.3 (Qiagen) with an in-house workflow (Fig. S1). Imported raw reads were trimmed for quality and adaptor sequences after the initial data QC. Subsequently, four rounds of host sequence removal were applied against four swine genomes (GenBank assembly accession: GCA_002844635.1, GCA_000003025.6, GCA_015776825.1, and GCA_006511355.1). The remaining reads were *de novo* assembled. Assembled contigs were subjected to taxonomic profiling against the built-in Pathogen Reference Database (PRD)-virus or -bacteria and then verified by CLC BLASTn with a cutoff e-value ≤ e^-100^ against the built-in databases of ref_viruses_rep_genomes for all three libraries and ref_prok_rep_genomes for the dsDNA library. Due to limited bioinformatics computational power available, to reduce running time the strict e-value and PRD instead of nucleotide (nt) database were used in the BLASTn process. These settings as well as incorrect assembling and taxonomic profiling may cause false-negative errors. Therefore, the reads after host sequence removal were also subjected to CLC BLASTn with the same e-value against specially interested pathogen genomes of MRV, pseudorabies virus (Suid herpesvirus 1), porcine teschovirus, and porcine hemagglutinating encephalomyelitis virus that have been reported to be related to neurological diseases. Both contigs and reads that passed CLC BLASTn were subject to the final examination in NCBI BLASTn against the nt database to determine their taxonomic identification.

Import reads from Basespace

QC for Sequencing Reads

Trim Reads

Data QC and Clean Host DNA

Remaining reads

De Novo Assembly

Taxonomic Profiling against

PRD-virus or -bacteria

CLC BLASTn against ref_viruses_rep_genomes and ref_prok_rep_genomes databases

CLC BLASTn against specially interested genomes

NCBI BLASTn against nt database

**Fig. S1**. In-house workflow of sequencing data analysis. The last step of BLASTn was conducted with NCBI web BLAST. All the other steps were processed with CLC Genomics Workbench 21.0.3 (Qiagen).

**Results**

***Sensitivity and specificity of developed MRV real-time PCR***

A real-time PCR (RT-qPCR_L1) was developed by targeting the conserved region of MRV L1 gene in order to quantify virus loads in samples collected from the pig study. To determine the sensitivity of the developed RT-qPCR_L1 assay, the L1 gene of the MRV isolate was cloned into a T7 promoter vector and in vitro transcribed to produce viral RNA. The analytic sensitivity of RT-qPCR_L1 assay showed a detection limit of 10 RNA copies and with a cutoff of threshold cycle (Ct) of 40. Further analysis showed a linear correlation of a series of RNA dilutions with R^2^ higher than 0.999, indicating that this assay is reproducible and quantitative (data not shown). We also detected RNA samples extracted from the serially diluted MRV isolate with a known titer, and results showed that the assay was able to detect 100 TCID_50_ per mL of the MRV virus. In addition, swine pathogen DNA and RNA including from TGEV, PEDV, SIV and PRRSV were negative by the developed RT-qPCR_L1 assay using the MRV isolate as the positive control, indicating that the assay is specific. These results indicate that the developed RT-qPCR_L1 can be used to determine virus titer of samples collected in the pig studies.

***Sequences of viruses and bacteria detected in swine clinical tissue homogenate***

A total of 41- 60 million raw reads were obtained from the sequencing of the three libraries. The reads after trimming and host sequence removal were assembled into 23- 236 thousands of contigs. A total of 35, 48, and 30 contigs were identified as virus sequences after taxonomic profiling and CLC BLASTn at the cutoff of e-value ≤ e^-100^ in the RNA, ssDNA, and dsRNA libraries respectively, while 33 contigs in the dsDNA were bacterial sequences. The final NCBI BLASTn examination found 15 of them were porcine parvovirus, 3 were porcine adenovirus, and 31 belonged to the following 27 bacterial species: *E. coli, Prevotella copri, Planococcus plakortidis, Prevotella jejuni, Parabacteroides distasonis, Pseudobutyrivibrio xylanivorans, Streptococcus equinus, Staphylococcus haemolyticus, Lactobacillus delbrueckii, Shigella dysenteriae, Paracoccus zhejiangensis, Lactobacillus delbrueckii, Lactobacillus agilis, Shigella dysenteriae, Shigella sonnei, Prevotella copri, Psychrobacter pulmonis, Selenomonas ruminantium, Prevotella copri, Faecalibacterium prausnitzii, Paracoccus kondratievae, Actinobacillus porcitonsillarum, Salmonella enterica, Sphingomonas panni, Prevotella dentalis, Paracoccus liaowanqingii, Paracoccus aminophilus,* and *Ligilactobacillus salivarius*.

The reads after removing host sequences were searched with CLC BLASTn against specially interested virus genomes of as described above, and then also confirmed with BLASTn against the nt database to avoid false negative errors. It was found that one pair of reads (237 bp) in the RNA library belonged to MRV which is 100% sequence identity to the novel MRV/Porcine/USA/2018 isolate. This is consistent with the very low virus load present in the tissue sample, resulting in that it took 3 passages to isolate this MRV strain. Other viruses including pseudorabies virus (Suid herpesvirus 1), porcine teschovirus, and porcine hemagglutinating encephalomyelitis virus were not identified.


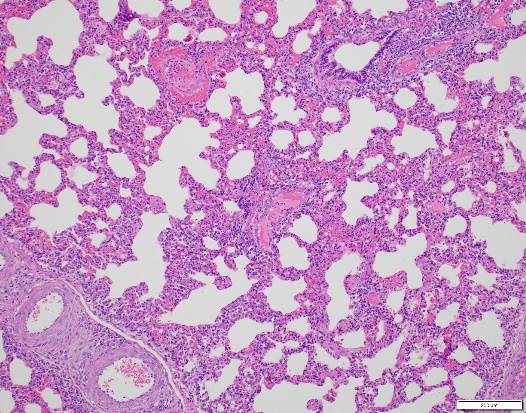

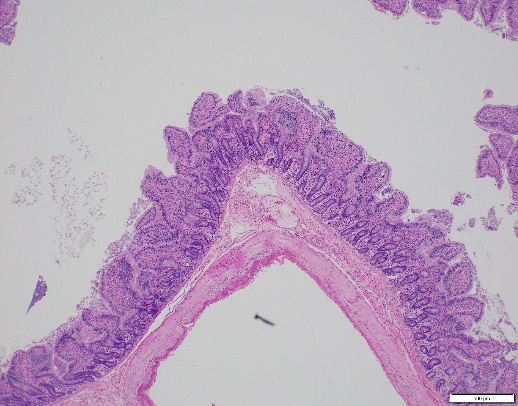

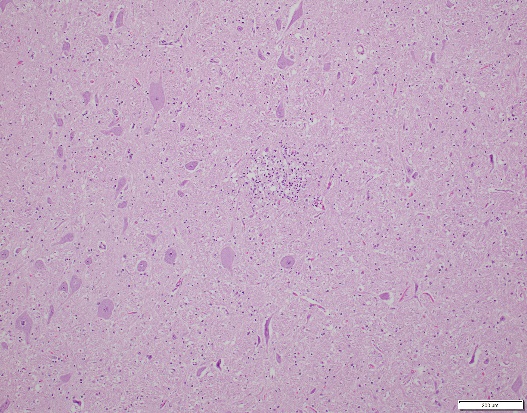

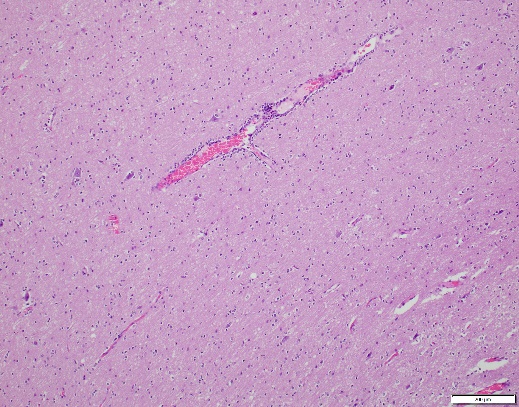


A)

B)

C)

D)

**Fig. S2**: Histopathological changes in the lung, intestine and brain of the pigs with neurological signs. A) There are mild to moderate mixed interstitial and perivascular inflammation with multi focal perivascular haemorrhage (Scale bar 200 µm). B) Mild edema and mixed inflammation in the lamina propria. The villi are contracted/blunt, and the submucosa has congested vessels and mild edema (Scale bar 500 µm). C) Brainstem gliosis is evidenced by focal area of gliosis and neuropil rarefaction in region of obex (Scale bar 200 µm). D) Brain perivascular is evidenced by mixed inflammatory infiltrates surrounding rare vessels. Mild neuropil vacuolation may be associated with edema or preservation artifact (Scale bar 200 µm).


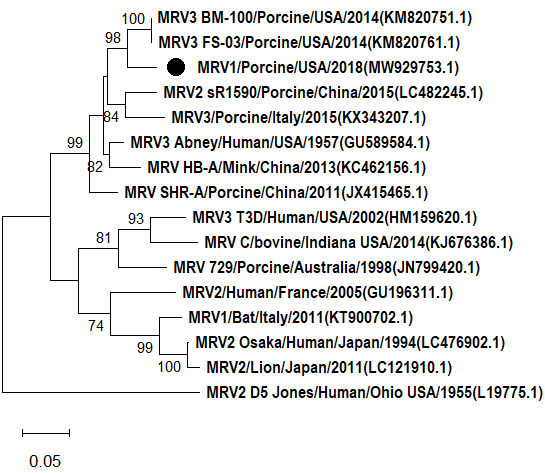

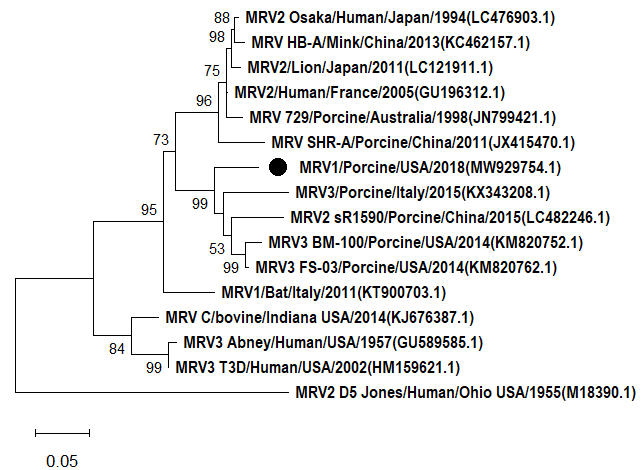


**C)**

**B)**

**A)**

S3

S2


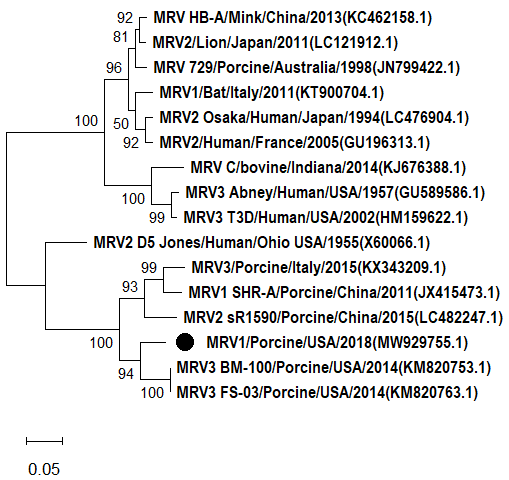

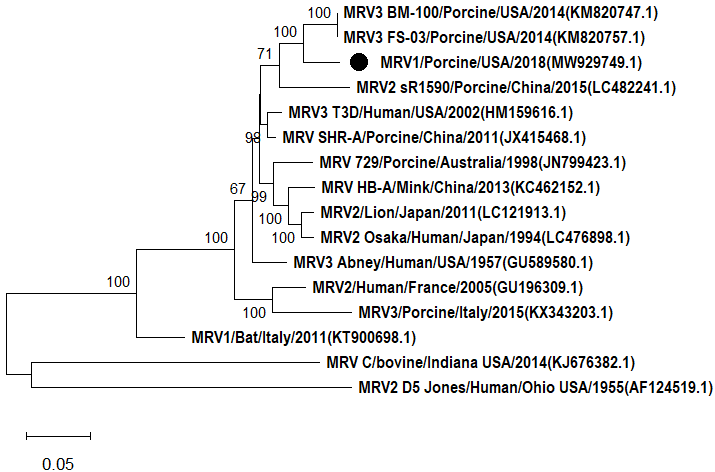


**D))**

S4

M1


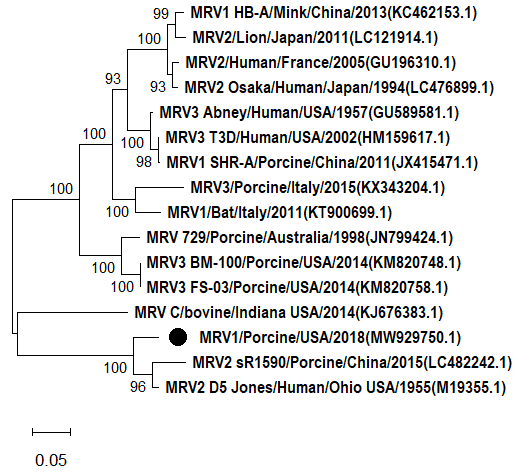

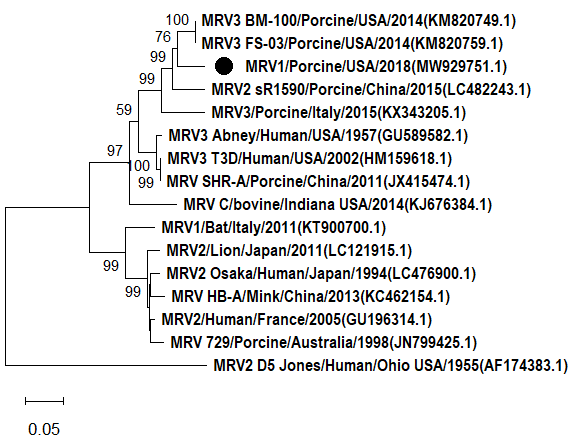


**H))**

**G))**

**E))**

**F))**

M3

M2


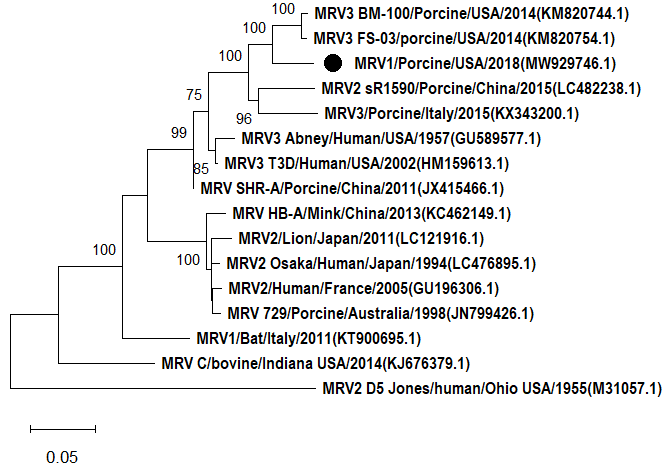

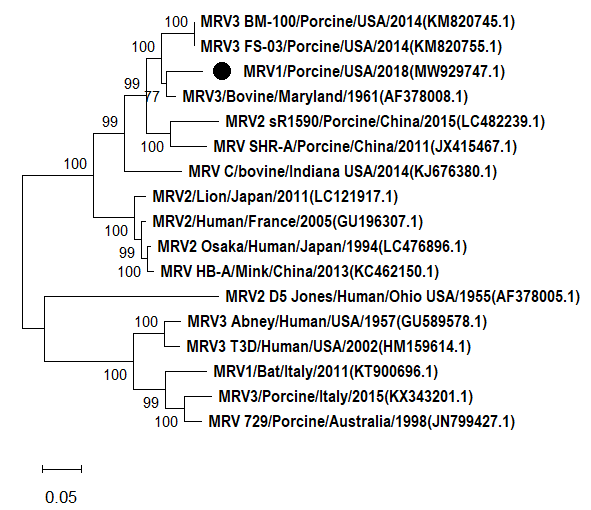


L2

L1


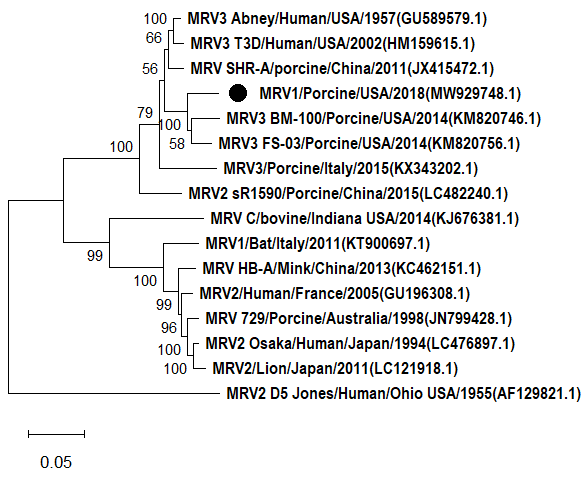


**I))**

L3

**Fig. S3. Phylogenetic trees of nine segments of the novel reassortant MRV/Kansas USA/Porcine/2018.** Related MRV strains were downloaded from GenBank, and open reading fame of each gene segment was used for building the phylogenetic trees. The MRV isolate identified in this study is labelled with a round dot. A): S2 segment; B): S3 segment; C): S4 segment; D): M1 segment; E): M2 segment; F): M3 segment; G): L1 segment; H): L2 segment; I): L3 segment.

**Supplemental Table 1. Primers and probe used in real-time RT-PCR assay**

|  | **Primers ⁄ probe sequence** | **Location of primers** | **Amplicon (bp)** |
| --- | --- | --- | --- |
| L1-probe | 5’-TGGCAGCGDTGGATACGTTATTC-3’ | 2521-2543 | 137bp |
| L1-F | 5’-GCGAAYTCTTCAGCRGAGGAGC-3’ | 2434-2455 |  |
| L1-R | 5’-CGTGARAAAGCACAGCATARAGCC-3’ | 2547-2570 |  |

| **dpi** | **# of infected pigs** | | | | | | | | | **# of contact pigs** | | |
| --- | --- | --- | --- | --- | --- | --- | --- | --- | --- | --- | --- | --- |
|  | **#20** | **#30** | **#60** | **#22** | **#29** | **#32** | **#21** | **#31** | **#61** | **#27** | **#49** | **#64** |
| **0 dpi** |  |  |  |  |  |  |  |  |  |  |  |  |
| **1 dpi** |  |  | Diarrhea |  |  |  |  | Diarrhea |  |  |  |  |
| **2 dpi** | Walking discordant |  |  | Fever |  | Fever  N⊕10^5.02^/ml |  | N⊕10^5.70^/ml | R⊕10^5.14^/ml |  |  |  |
| **3 dpi** | Fever  Walking discordant |  |  |  |  |  | R⊕10^6.16^/ml |  | Fever |  | Fever |  |
| **4 dpi** | Fever  Walking discordant  H&E⊕  IHC － | Fever diarrhea  ileum10^7.93^/g jejunum10^7.69^/g colon10^6.8^/g ⊕  H&E⊕ IHC ⊕ | N⊕10^5.25^/ml  duodenum 10^5.2^/g⊕  H&E⊕ IHC ⊕ | Fever  Diarrhea  R⊕10^5.95^/ml | Fever | Fever | R⊕10^6.31^/ml | R⊕10^6.5^/ml | Fever  R⊕10^5.47^/ml |  |  |  |
| **5 dpi** | Necropsy at 4 dpi | | |  |  | Fever | Diarrhea |  | Fever  R⊕10^5.32^/ml |  |  | Diarrhea |
| **6 dpi** |  |  |  |  | Fever  Nasal discharge | Nasal discharge |  |  |  | Nasal  discharge |  |  |
| **7 dpi** |  |  |  | Diarrhea  HI: 40 | Fever  Nasal discharge  HI: 40 | Nasal discharge  HI: 80 | Diarrhea  HI: 320 | HI: 80 | Diarrhea  HI: 40 | Fever  Nasal  discharge |  | Diarrhea |
| **8 dpi** |  |  |  | Necropsy at 7 dpi | | | Diarrhea |  |  |  | Diarrhea |  |
| **9 dpi** |  |  |  |  |  |  | Diarrhea  HI:160 | HI: 80 | HI: 80 |  | Diarrhea  Colon10^7.82^/g⊕ | Diarrhea  Duodenum10^5.32^/g |
| **10 dpi** |  |  |  |  |  |  |  |  |  | Necropsy at 7 dpc (9 dpi) | | |
| **11 dpi** |  |  |  |  |  |  |  |  |  |  |  |  |
| **12 dpi** |  |  |  |  |  |  |  |  |  |  |  |  |
| **13 dpi** |  |  |  |  |  |  |  |  |  |  |  |  |
| **14 dpi** |  |  |  |  |  |  | HI: 320 | HI:80 | HI:320 |  |  |  |
|  |  |  |  |  |  |  | Euthanasia at 14 dpi | | |  |  |  |

**Supplemental Table 2. Summary of pig study result of each infected and contact pig**

**Note**: “N” means nasal swabs; “R” means rectal swabs; “⊕” means positive; “－” means negative.
